# Supplementary material for: Fatty Acid Excess Dysregulates CARF to Initiate the Development of Hepatic Steatosis
Source: Cells. 2023 Apr 1;12(7):1069. doi: 10.3390/cells12071069 (PMC10093423; doi:10.3390/cells12071069)
Supplement: Supplementary file 1 [file cells-12-01069-s001.zip › cells-2237429-Supplementary.pdf]

cells MDPI

Supplementary Materials

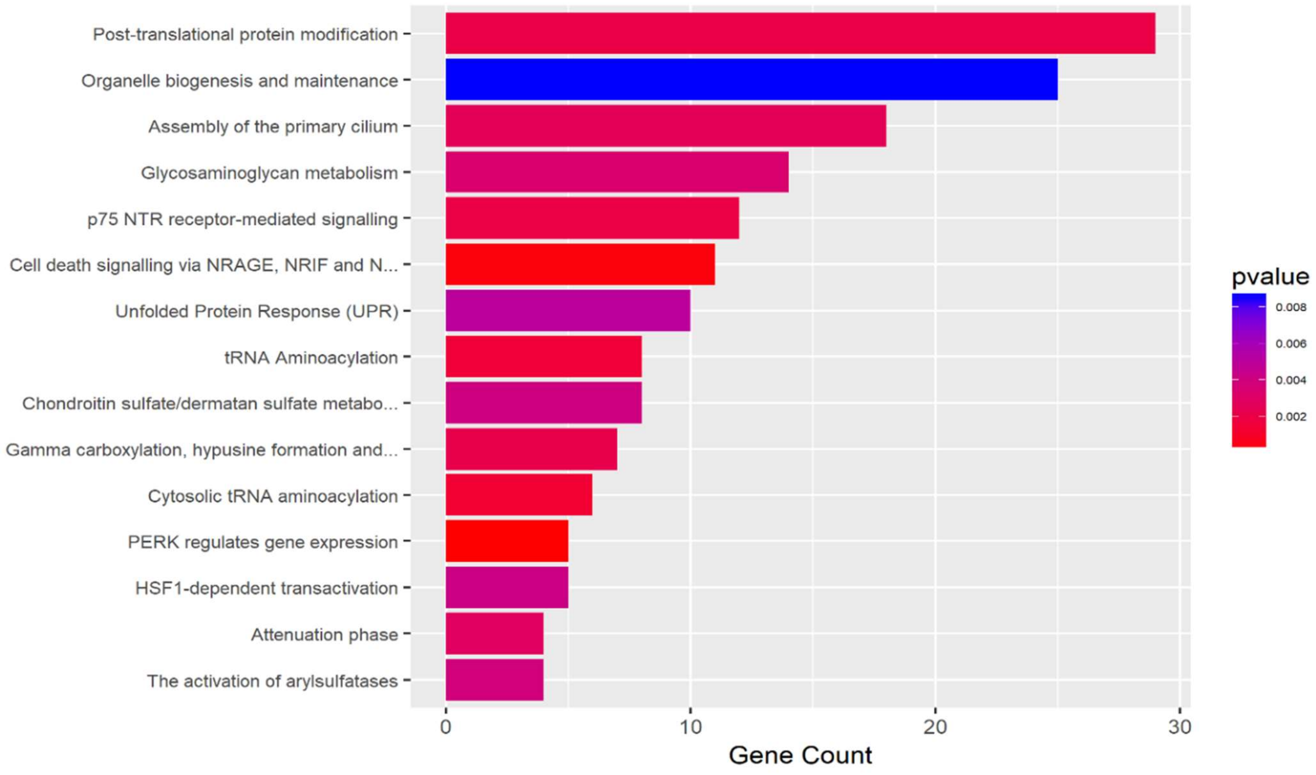

Figure S1. Reactome pathway analysis. The Reactome pathways enrichment analysis of differentially expressed genes in CARF depleted HepG2 cells.

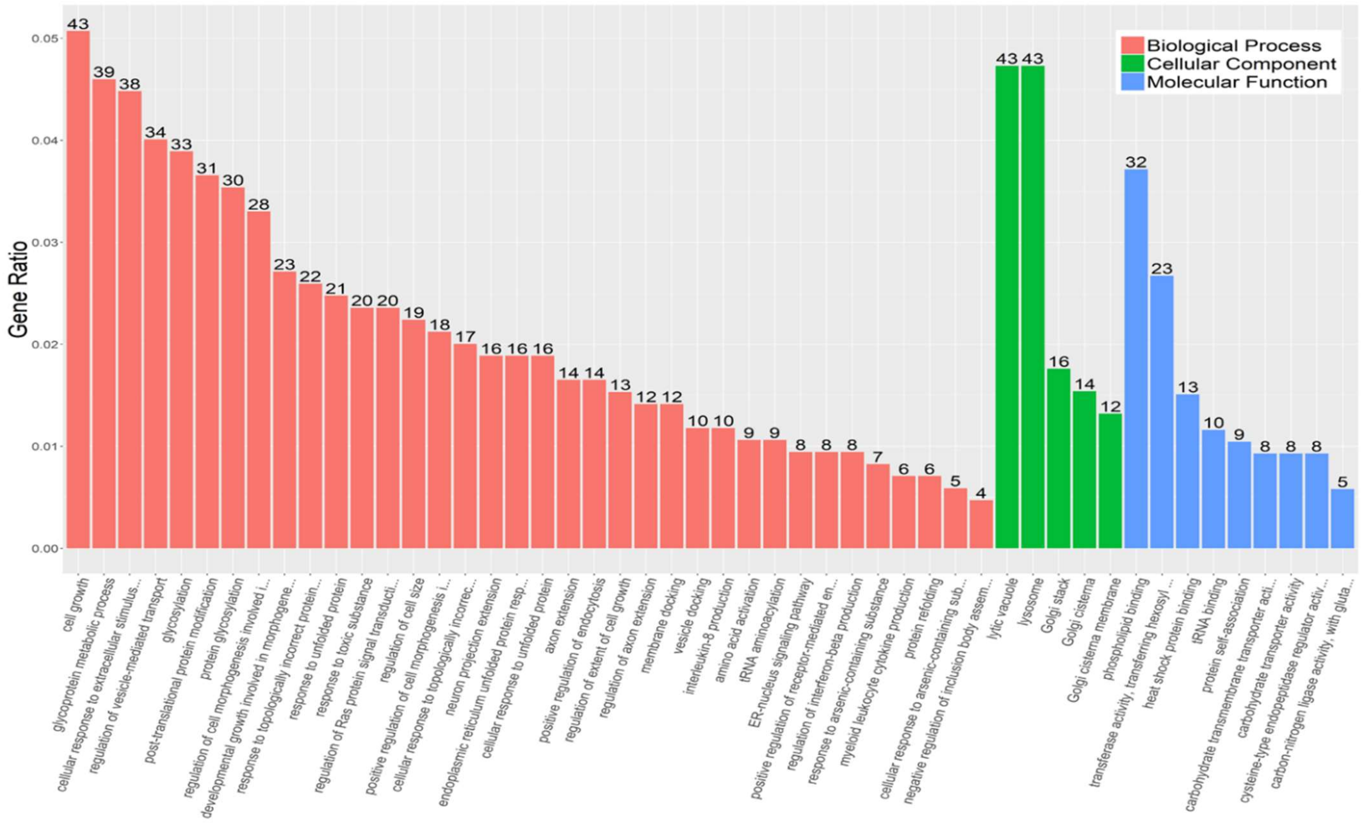

Figure S2. Gene Ontology analysis. Gene ontology enrichment analysis of biological processes for differentially expressed genes, either up regulated or downregulated, compared between Sh-scramble RNA and Sh-CARF RNA treated HepG2 cells.

Table S1. List of ER stress associated differentially expressed genes.

Gene symbol	NAME	Fold change	pValue
ATF5	activating transcription factor 5	1.62	3.63E-09
ATF6	activating transcription factor 6	1.41	5.36E-15
DDIT3	DNA-damage-inducible transcript 3. (CHOP)	1.97	2.55E-42
DNAJB9	DnaJ (Hsp40) homolog; subfamily B; member 9	1.42	3.63E-10
EDEM1	ER degradation enhancer; mannosidase alpha-like 1	1.52	1.16E-14
ERN1 (IRE1a)	endoplasmic reticulum to nucleus signaling 1	1.60	3.05E-14
HSPA14	heat shock 70kDa protein 14	1.34	5.25E-09
HSPA1A	heat shock 70kDa protein 1A	1.78	3.76E-07
HSPA1B	heat shock 70kDa protein 1B	1.68	2.50E-06
HSPA4	heat shock 70kDa protein 4	1.42	1.33E-22
HSPA5	(BiP) (GRP78 (glucose-regulated protein; 78kDa)	1.50	4.44E-18
HSPA6	heat shock 70kDa protein 6 (HSP70B')	1.74	6.64E-08
HSPA8	heat shock 70kDa protein 8	1.92	3.91E-27
HSPA9	heat shock 70kDa protein 9 (mortalin)	1.75	5.00E-62
NUPR1	nuclear protein; transcriptional regulator; 1	2.00	5.93E-84
TRIB3	tribbles pseudokinase 3	1.43	1.40E-2
CTH	cystathionine gamma-lyase	1.93	3.34E-07
CUL7	cullin 7	-1.72	1.24E-38
CREB3L3	cAMP responsive element binding protein 3-like 3	1.92	1.09E-32
UGGT1	UDP-glucose glycoprotein glucosyltransferase 1	-1.59	1.09E-18

Ca2+ Signaling associated genes

CAMKK2	calcium/calmodulin-dependent protein kinase kinase 2; beta	-1.39	1.03E-10
ERLIN2	ER lipid raft associated 2	-2.76	1.87E-61

WFS1	Wolfram syndrome 1 (wolframin)	-2.31	1.15E-62
ATP2B1	ATPase, Ca++ transporting, plasma membrane	1.61	2.51E-28
ATP2B4	ATPase, Ca++ transporting, plasma membrane 4	-1.78	2.23E-05
MBTPS2	membrane-bound transcription factor peptidase, site 2 -	1.55	1.55E-08

PERK regulated genes

ATF3	activating transcription factor 3	1.52	2.40E-27
ASNS	asparagine synthetase (glutamine-hydrolyzing)	2.83	8.73E-146
DDIT3	DNA-damage-inducible transcript 3	1.97	2.55E-42
HERPUD1	homocysteine-inducible, endoplasmic reticulum stress- inducible, ubiquitin-like domain member 1	1.67	6.10E- 52
IGFBP1	insulin-like growth factor binding protein	1.77	4.28E-42

Table S2. List of PCR primers.

Gene name	Orientation	Sequences (5′-3′)
CARF	Forward	TCATCTCCTTTTCCAT GGCC
	Reverse	TCTTGGCAACCAGTTC ATCT
ERN1	Forward	CACAGTGACGCTTCCTGAAAC
	Reverse	GCCATCATTAGGATCTGGGAGA
HSPA1A	Forward	CGCAACGTGCTCATCTTTGA
	Reverse	TCGCTTGTTCTGGCTGATGT
HSPA5	Forward	CATCACGCCGTCCTATGTCG
	Reverse	CGTCAAAGACCGTGTTCTCG
HSPA6	Forward	GATGTGTCGGTTCTCTCCATTG
	Reverse	CTTCCATGAAGTGGTTCACGA
18S-RNA	Forward	GAGGTGAAATTCTTGGACCGG
	Reverse	CGAACCTCCGACTTTCGTTCT
